# Air–Noise Pollution Linkages: Testing Innovative Community-Based Adaptation and Mitigation Strategies in Kenya

**DOI:** 10.5334/aogh.4750

**Published:** 2025-10-28

**Authors:** Manasi Kumar, Ngongang Wandji Danube, Vincent Nyongesa, Lucas Kalama, Carol Ngunu, Hassan Leli, Albert Tele, Edith Apondi, Josphat Asande, Osman Warfa, Ayub Macharia, Beatrice Madeghe, Obadia Yator, Darius Nyamai, Philip Osano

**Affiliations:** 1 Department of Population Health and the Institute for Excellence in Health Equity, New York University Grossman School of Medicine, NY, USA; 2 Stockholm Environment Institute, Africa office, Nairobi, Kenya; 3 Department of Psychiatry, University of Nairobi, Nairobi, Kenya; 4 Kilifi Youth Advisory Champion for Health, Kilifi, Kenya; 5 Nairobi County Health Directorate, Nairobi, Kenya; 6 Kilifi County Health Directorate, Kilifi, Kenya; 7 Department of Clinical Developmental Psychology, Vrije University, Amsterdam, Netherlands; 8 Kariobangi North Health Center, Nairobi, Kenya; 9 Kangemi Health Center, Nairobi, Kenya; 10 Ministry of Health, Nairobi, Kenya; 11 National Environment Management Authority, Nairobi, Kenya; 12 Department of Medical Research, Kenyatta National Hospital, Nairobi, Kenya; 13 School of Mental Health, Defence College of Health Sciences, National Defence University, Kenya; 14 Nairobi City County Government, Nairobi, Kenya; 15 CIFOR-ICRAF, World Agroforestry Centre, Nairobi, Kenya

**Keywords:** environmental determinants, air quality, monitoring, noise pollution, perinatal adolescents, mental health

## Abstract

*Introduction:* Our case study was conducted across healthcare facilities in Kilifi and Nairobi, where perinatal adolescents were screened for depression.

*Objective:* The relationship of environmental monitoring in addressing mental health needs of vulnerable perinatal adolescent populations was explored.

*Methods:* We installed outdoor air quality sensors at two facilities in Nairobi—Kangemi and Kariobangi North health centers—and two in Kilifi—Mtwapa and Vipingo health centers—and installed sensors in two households of two perinatal adolescents. Community health workers monitored air quality and noise levels data, collecting experiential data on stress and mood from perinatal adolescents.

*Findings:* Air quality monitoring revealed site-specific variations in PM_2.5_ concentrations. Kariobangi Health Center recorded the highest mean concentration of 29.45 µg/m³, exceeding the WHO 2021 annual guideline of 5 µg/m³ indicating substantially degraded air quality. Kangemi Health Center was next (21.27 µg/m³), followed by Mtwapa (15.34 µg/m³) and Vipingo (12.52 µg/m³). Noise monitoring revealed consistently elevated exposure in healthcare settings. At Kangemi Health Center, mean noise levels reached 52.2 dB (median: 53.5 dB), surpassing the WHO guideline for hospital settings (<35–40 dB). Household-level air quality monitoring highlighted significant operational challenges: sensor deployment constraints, difficulties in ensuring continuous temporal coverage, and substantial intra-day variability—underscoring the need for improved monitoring design and calibration strategies.

*Conclusions:* We tested air and noise monitoring deployment as a lever for strengthening the health system and a strategy for improved patient care and mental well-being. We trained community health workers and youth leaders in a task-shifting model to collect environmental health data. Our approach sought to ease the deployment of environmental monitoring in a sustainable data collection process. However, both mitigation, targeting reduction in sources of pollution, and adaptation efforts focused on coping with the effects of air and noise pollution on vulnerable populations within primary care need concerted efforts.

## Background

### Mental health and environmental considerations for perinatal adolescent populations

The perinatal period represents a challenge for women’s mental health for several reasons. Factors impacting perinatal mental health include role adjustments, hormonal changes, body shape, and possible complications during pregnancy, childbirth, or postpartum [1]. Climate change is the greatest threat to global public health [2]. In 2019, approximately 24.9 million people worldwide were displaced due to extreme weather; without quick intervention, this number is expected to grow to 200 million annually by 2050 [3]. Global temperature increases and destruction of the natural world are harming the overall health of populations at large [4]. Increase in temperatures has caused increased dehydration and renal function loss, dermatological malignancies, tropical infections, adverse mental health outcomes, pregnancy complications, allergies, cardiovascular and pulmonary complications, morbidity, and mortality [2, 5]. Climate change’s physical and mental health effects greatly impact perinatal adolescents [6]; however, effects on this group have not been sufficiently understood [7]. Food insecurity and adverse social conditions resulting from climate change further aggravate the well-being of perinatal girls and women [8]. The negative impact of climate change on educational attainment undermines socioeconomic prospects of girls, further compromising their well-being, especially sexual and reproductive health and rights [6, 9]. Extreme weather events such as extreme heat, floods, droughts, and pollution cause economic costs and loss of human lives [4, 10, 11]. Evidence shows an association between extreme weather event-related maternal stress and poor child health outcomes [12–14]. Maternal stress during disaster has been linked to childhood adiposity [12, 13, 15], poorer infant temperament [14], impaired motor or cognitive development [16–19], and stunting [20]. A longitudinal birth cohort study that followed up into adulthood (*n* = 9065) participants with mental health data found that higher exposure to fine particulate matter (PM_2.5_) in pregnancy and childhood was associated with increased psychotic experiences, and in pregnancy was associated with higher rates of depression [21]. Higher noise pollution exposure in childhood and adolescence was associated with increased anxiety [21]. Adolescents and young perinatal women need to be educated on environmental pollution and its impact on their health and that of their children.

### Leveraging community health and primary healthcare workers to advance the environmental health agenda

Community healthcare workers (CHWs) interact with households regularly; equipping them with environmental health information and intervention tools is critical in taking the message to the household level. Primary healthcare clinicians need to focus not only on treating diseases but also on paying attention to and checking patient environmental aspects that could contribute to diseases. Healthcare professionals should recognize the potential impacts that environmental disruptions can have on young women during pregnancy, and their awareness of the impacts can ensure that appropriate treatment and safety measures are implemented [15]. Mental health impacts of climate change may be overlooked by healthcare providers who do not recognize risks or know how to respond to these impacts [22]. Birth professionals, midwives, and providers within primary care clinics have an essential role in ensuring mental health and well-being are emphasized in this increasingly climate-changed era [23].

### Routine monitoring of adverse environmental exposures

Environmental health monitoring has three major components: hazard monitoring, exposure monitoring, and health outcome (health effects) monitoring [24]. Routine monitoring of the environmental health of communities and public health facilities can be instructive in understanding and responding to adverse exposures like poor air and water quality. Knowledge of measures and their timely deployment could help ensure that perinatal women and their infants minimize chances of developing complications arising from harmful environmental toxins [25]. Installing air quality sensors at health facilities and households could help monitor air quality. The World Health Organization (WHO) defines environmental health indicators as those that imply monitoring, action, and advocacy [26]. WHO also defines these environmental health indicators as domains of information ‘about a scientifically based linkage between environment and health’; thus, it is critical for a relevant and meaningful indicator to capture the interconnection between environment and health—ideally, a health outcome with an environmental causation [26]. To carry out routine health monitoring, these indicators must be simple, measurable, understandable, and defensible [24].

Our study explored whether youth and CHWs can feasibly implement environmental monitoring to inform perinatal adolescent mental health interventions in Kenya. We present process findings^1^ of air quality and noise indicators that formed part of a routine environmental health outcomes monitoring study piloted at select households and health facilities. This study ascertained the general feasibility and acceptability of environmental exposures’ assessment and the impact of routine monitoring of air and noise pollution on perinatal adolescent health in Kenya.

## Approach

### Settings

The work was conducted in two primary healthcare facilities from Kilifi County, and two from Nairobi County. Sensors were installed in two households in Kilifi, and their successful deployment and use were studied.

#### Kariobangi and kangemi health centers in nairobi

Kariobangi and Kangemi health centers are level 3 facilities under the Nairobi County Council. A level 3 facility includes health centers, maternity, and sub-district hospitals that are more equipped than level 1 and level 2 facilities and slightly less equipped to handle advanced specialist care like level 4 hospitals. Kariobangi consists of lower-middle-class areas and slum-type regions (Kariobangi North) [27]. Kangemi Health Center is located in a slum in Nairobi City within a small valley on the city’s outskirts.

#### Mtwapa hospital and vipingo health center in kilifi

Mtwapa hospital is a level 4 facility located in Kilifi County, Kilifi South Constituency, Kilifi South Sub-County, and Shimo La Tewa ward. Vipingo health center is a level 3 facility located in Kilifi County, Kilifi South Constituency, Kilifi South Sub-County, and Junju ward.

#### Participants

The study involved facility in-charges (*n* = 4) from the four facilities, CHWs and youth leaders (*n* = 10), and county officers (*n* = 8), including those leading environmental and adolescent health, who were invited and updated throughout the work. Two research assistants (RAs), one in Nairobi (VN) and one in Kilifi (LK) oversaw deployment and monitoring with two CHWs per site. RAs were trained by an engineer and environmental exposure specialist from the Stockholm Environment Institute (SEI) Africa office located in Nairobi, Kenya, which was a technical partner in this study. SEI-Africa loaned air and noise pollution sensors to health facilities under this study (see Box 1).

**Box 1. Sequence of Activities Involved in the Placement of Sensors**

**Table:** 

Site visits and field deployment discussion (May–June 2023)
Training of research assistants, community health volunteers, and youth leaders, meeting health facility heads for permissions (March–June 2023)
Identification of the right sensors and their deployment (June 2023)
Survey with pregnant and parenting adolescents, interviews with health workers, adolescents, and policymakers (not reported in this paper, but findings reported separately), July–September 2023
Journey mapping and ecological momentary analysis on mental health and general health in three participants (June–November 2023)
Post-deployment training and follow-up (July 2023–May 2024)
Continuous testing and feedback (July 2023–June 2024)

### Tools and instruments

IQAIR AirVisual outdoor air quality monitor provides accurate, configurable, and continuous air monitoring in covered areas outdoors. It measures temperature, humidity, pressure, carbon dioxide (CO_2_), and particulate matter. Power can be supplied with the PoE injector (Power Over Internet) and included cable via the optional USB adapter. Connections to the internet are made via Ethernet cable, Wi-Fi, or a USB 4G modem. It supports multiple power and network options [28].

Blood pressure was monitored using a blood pressure machine. We asked about self-reported stress^2^ using a 5-point Likert scale (1 being least stressed or good mood and 5 being high stress and depressed mood). We asked about their mood^3^ levels using a mood rating scale, while pulse, heart rate, and blood oxygen were measured using a wristband. Carbon monoxide (CO), CO_2_, formaldehyde (HCHO), air quality index (AQI), and total volatile organic compounds (TVOC) were measured by a portable air quality sensor.

**Figure 1:**
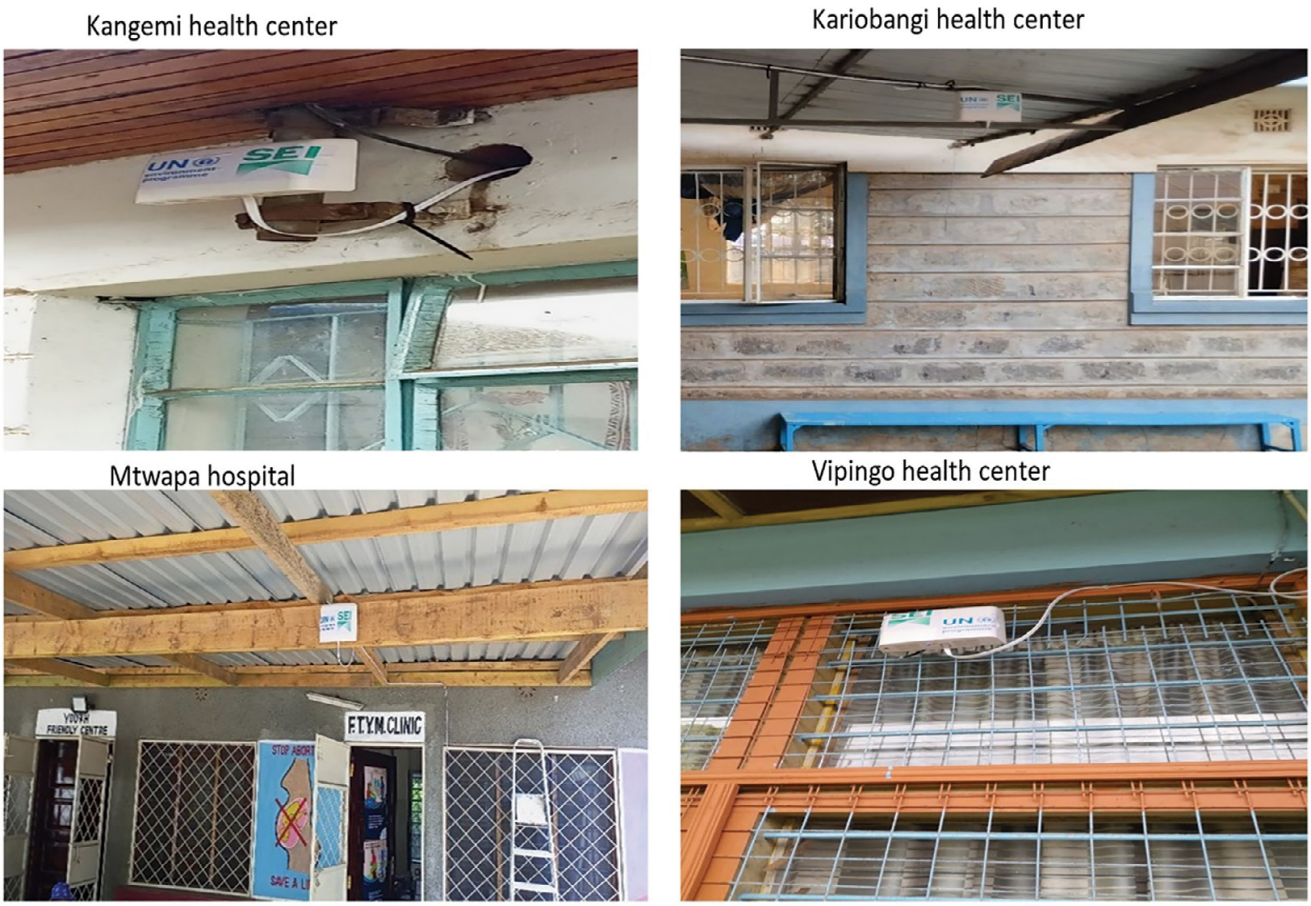
Outdoor air quality sensors at health facilities, showing where they were installed and positioned.

*Facility-level deployment of air quality and noise sensors:* The health facilities had internet connectivity using Wi-Fi internet for Mtwapa hospital and Vipingo health center, and Ethernet ports for Kariobangi and Kangemi. The data were collected from air quality sensors via any available internet connectivity to the IQAir AirVisual Cloud [28], which processes and distributes the data to the app and TV screens. Outdoor air quality sensors were installed in June 2023 in all four health centers in Nairobi and Kilifi (see Figure 1).

**Figure 2:**
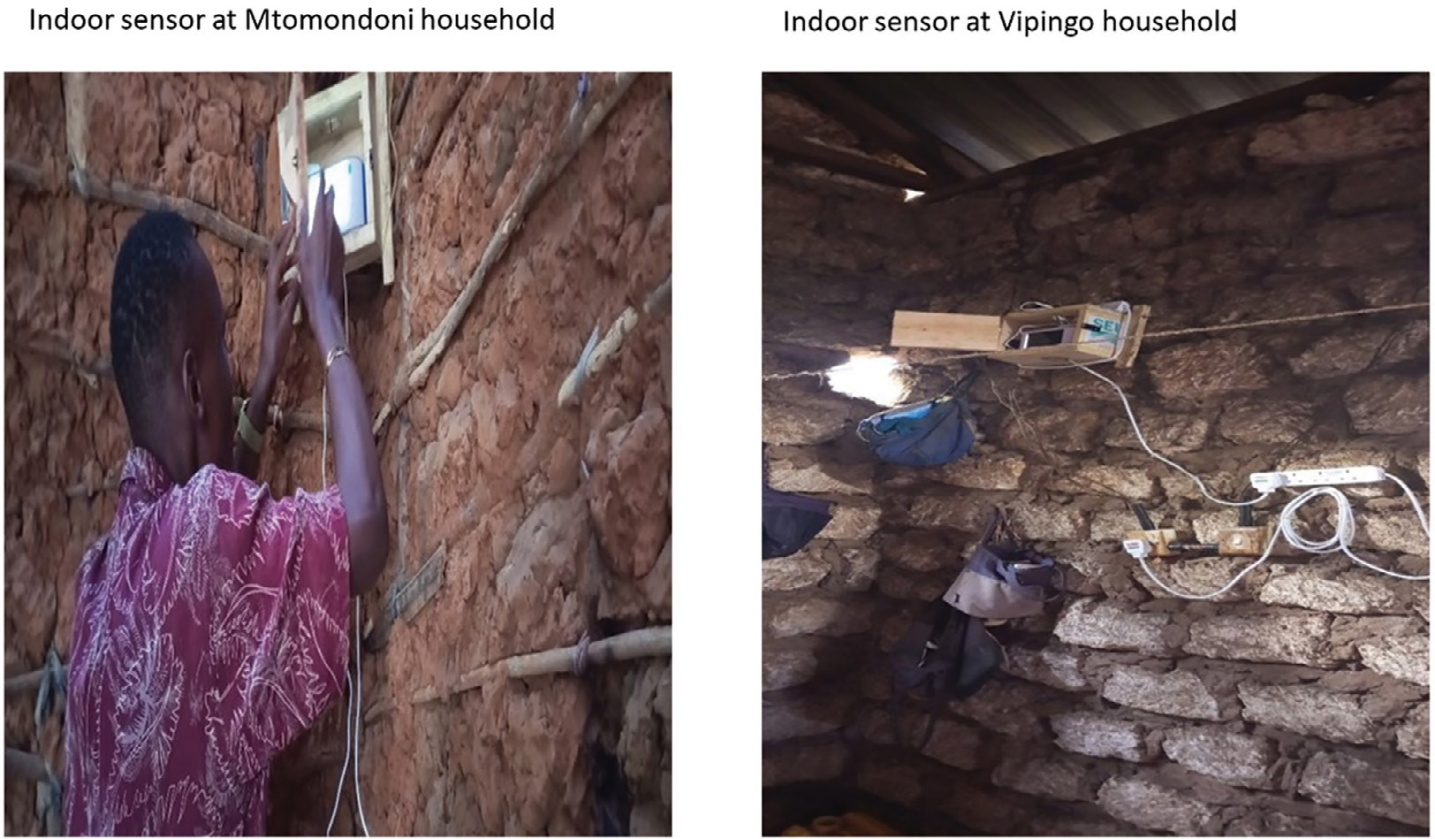
Indoor sensors in households, showing their household infrastructure and sensor placement.

*Household sensor deployment:* Indoor air quality sensors (with the capacity to capture both indoor and outdoor conditions) were deployed in two households in Kilifi—one located near Mtwapa Hospital and the other in proximity to Vipingo Health Center (see Figure 2). Household selection was guided by the availability of electricity and the presence of perinatal adolescents who had previously participated in the survey. The Mtwapa site was situated in the Mtomondoni Scheme, approximately 15–20 minutes by road from the hospital. However, after two months, the sensor had to be relocated to another adolescent’s household following marital discord and domestic violence due to which she changed residence. At Vipingo, the sensor was installed 500 meters from the health center after efforts to identify an alternative participant household with reliable electricity. Given the vulnerability of the selected households —including fragile structures, leaking roofs, and concerns over safety and security—custom protective wooden boxes with ventilation openings (~8 cm in diameter at the top and bottom) were securely mounted on walls to house the sensors. Internet connectivity was established using mobile routers (called MiFis) to ensure continuous data transmission.

*Household ecological momentary analysis exercise:* Data were collected from two participants whose households had indoor sensors over eight months in Kilifi. We asked participants to provide feedback on their mood and stress.

*Journey mapping:* A journey mapping exercise with two adolescents (aged 17 and 19 years) covering the Kariobangi North area was conducted, giving them a wristwatch, a blood pressure machine, and a portable air quality sensor. We sought their feedback on how they felt crossing places where we anticipated high noise and air pollution but were areas they visited regularly. For participant 1, point 1 was a playground she used to pass while going to school, behind the Kariobangi health center. Point 2 was the entrance of her school. Point 3 was an open roadside dumping place 50 meters away from her house. For participant 2, point 1 was approximately 100 meters from her house and next to the main road leading to her school. Point 2 was along a busy tarmacked road leading to her school. Point 3 was the entrance of her school, between residential houses. Point 4 was the entrance of Kariobangi health center, where she sought health services. Supplementary eFigure 1 shows the track followed by participant 2.

#### Noise sensor installation in kilifi county sites

In August 2023, the SEI staff trained a research assistant and a nurse at Mtwapa hospital on operating the sensor. A suitable location for installing the noise sensor was settled in the immunization area. Installation was done in a protective, closed wooden box with an open hole to allow the device to capture the noise. At Vipingo health center, installation was done in September. The research assistant identified an appropriate area to place the sensor behind the laboratory.

#### Noise sensor installation at nairobi county sites

**Figure 3:**
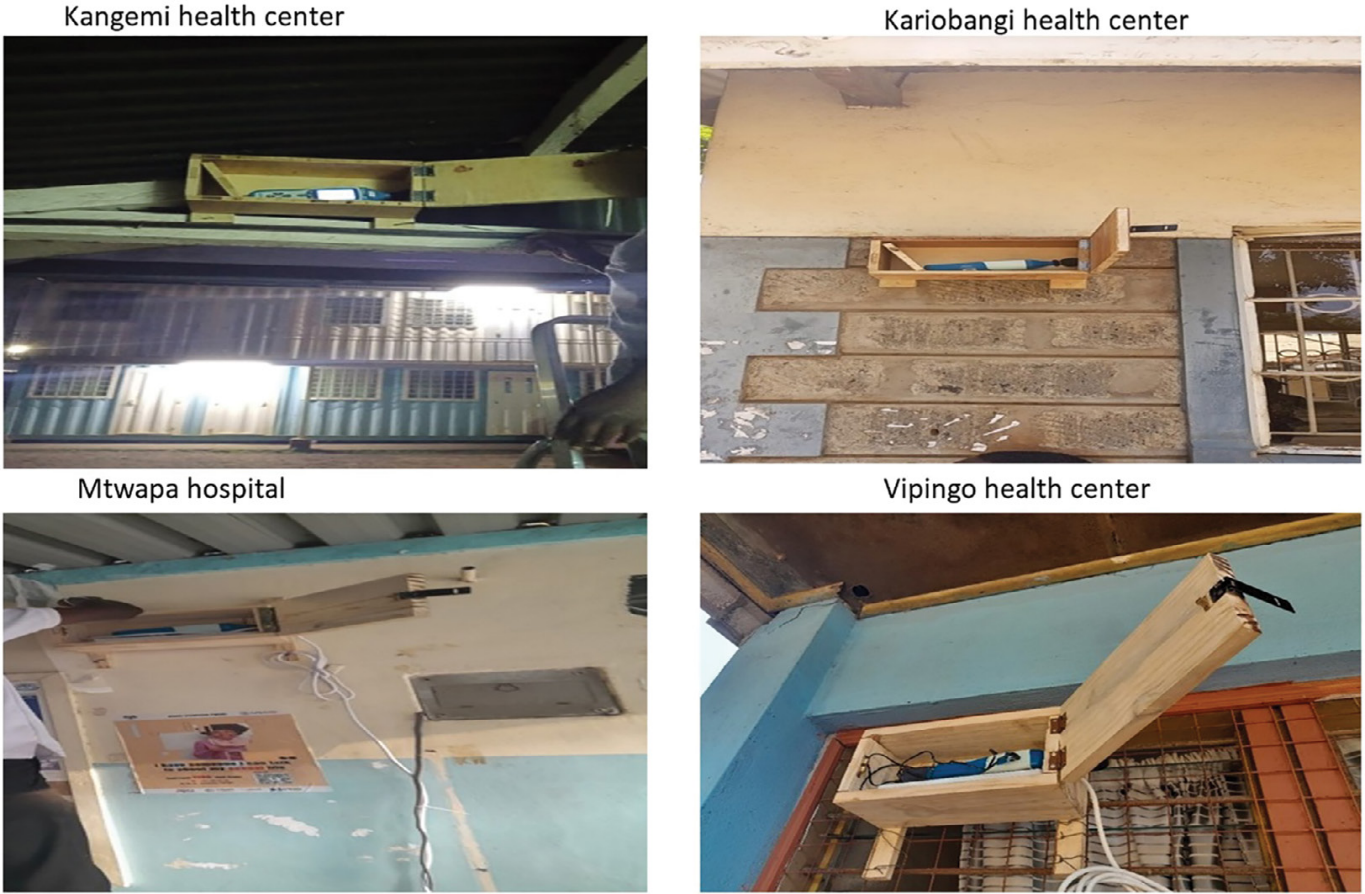
Noise sensors at health facilities, showing their placement.

Pretesting at Kariobangi North and Kangemi health centers was conducted in August and September 2023, respectively. At Kariobangi, it was installed in front of the antenatal care building, facing the main entrance and road, while at Kangemi, it was installed at the tuberculosis clinic, facing the main entrance and road. (see Figure 3).

## Data Collection and Analysis

### Training of health workers, youth leaders, and facility workers

In May 2023, healthcare workers in Kilifi (nurses, clinicians, and public health officers) were trained on mental health screening using the Mental Health Gap Action Programme (mhGAP) [29]. This was linked to the broad objective of studying environmental health factors that would impact the mental health of perinatal adolescents. They were trained on depression, psychological first aid, mental health assessments, nutrition, human participants’ protection, data collection principles, and tablet-based software. The parent study focused on mental health intervention for perinatally depressed adolescents by training facility-based staff in screening, management, and referral for mental disorders, namely depression, and using a stepped care approach by training CHWs in the delivery of group interpersonal psychotherapy [30, 31].

Survey participants were identified at antenatal and child welfare clinics and referrals from households within the communities. Eligibility screening was conducted by healthcare workers trained in mhGAP depression identification, and those who met eligibility criteria, being perinatal adolescents (ages 13–24 years) with an EPDS cut-off score of 10 and above, were invited to complete a mental health and environment survey conducted at the health facility by CHWs on two separate days.

Air quality measurements from health facility sensors were recorded between July 2023 and December 2023. Noise sensors were deployed across the four facilities, but usable data were successfully captured at only one site—Kangemi Health Center—where monitoring was conducted continuously for eight months (October 2023–May 2024)—measurements taken at a fixed point within the facility. Data collection at the remaining facilities was not feasible due to failures in the data transmission software and the absence of reliable desktop computers to transfer data from noise sensors.

Household air quality data were transmitted to a server hosted at SEI in real time. Complementary data using an ecological momentary analytic approach, assessing stress and mood, were collected by a youth leader and a CHW at different times of the day. However, due to a limited number of available devices, it was impossible to install household sensors in Nairobi, despite having mapped and identified potential sites (see Table 1). Additional methodological detail is provided in the supplementary document.

**Table 1:** Data collection unit and exposure studied.

DATA COLLECTION UNIT	EXPOSURE STUDIED	NATURE OF DATA CAPTURED
Health facility-level data	Indoor and outdoor air quality Noise pollution sensors	Continuous data captured from June to December 2023 Continuous data was captured from September 2023 to April 2024
Household-level data	Indoor and outdoor air quality	Failed in capturing data reliably due to numerous challenges
Adolescent participant-level data	Surveys and interviews on exposures, health conditions, mental health, and quality of life covering the Edinburgh Postnatal Depression Scale [32], Kessler’s 10 [33], Cohen’s Perceived Stress Scale [34], Generalized Anxiety Scales-7 [35], NIH PROMIS family functioning measures [36].	*N* = 241 survey data *N* = 40 qualitative data

Journey mapping was conducted with two adolescent mothers in Nairobi in April 2024. The exercise was disrupted by heavy rainfall and flooding, which rendered the selected routes impassable. Journey mapping is a qualitative research method that creates a visual timeline of a user’s experience with a product or service to highlight their actions, emotions, and pain points [37, 38]. Ecological Momentary Assessment (EMA) is a quantitative method that collects real-time data from users in their natural environment via technologies like smartphones or wearables [39]. When used together, they provide a powerful mixed-methods approach for user research and experience design.

Air quality assessment was based on PM_2.5_ exposure guidelines provided by the Kenyan Ministry of Environment and UNEP. We also summarize survey findings on environmental determinants of mental health. Journey mapping incorporated biomarker data from portable devices and ethnographic observations of the routes typically used by adolescent mothers.

### Ethical approvals

The study was part of a recently completed FIC grant (K43TW010716-05S1) focused on implementing mental health interventions for adole*s*cents in primary care LMIC cont*e*xts (INSPIRE). Ethical approval was sought from KNH/UoN ethics board (P913/12/2022) and the Kenyan scientific research approval via NACOSTI permit (NACOSTI/P/23/26253).

## Results

*Air quality in health facilities:* Urban-based health facilities exhibited poorer air quality than rural facilities. Household-level monitoring presented multiple logistical and contextual challenges. Electricity instability significantly constrained sensor deployment and data continuity. Additionally, limited awareness among participants regarding the function and purpose of sensors created hesitancy and resistance to installation. The living conditions—(as explained above)—further complicated deployment. These constraints led the research team to prioritize facility-based monitoring, providing representative insights into surrounding residential environments.

*Air quality in Nairobi households:* Initial steps in Nairobi included community engagement, training, and identifying suitable households for sensor placement. However, several barriers prevented implementation. These included unreliable power supply, heightened risks of sensor theft or damage in densely populated informal settlements, and mistrust among residents who questioned whether the devices might be used for surveillance. For these reasons, the study team discontinued household-level monitoring in Nairobi and concentrated exclusively on facility-based deployment.

**Figure 4:**
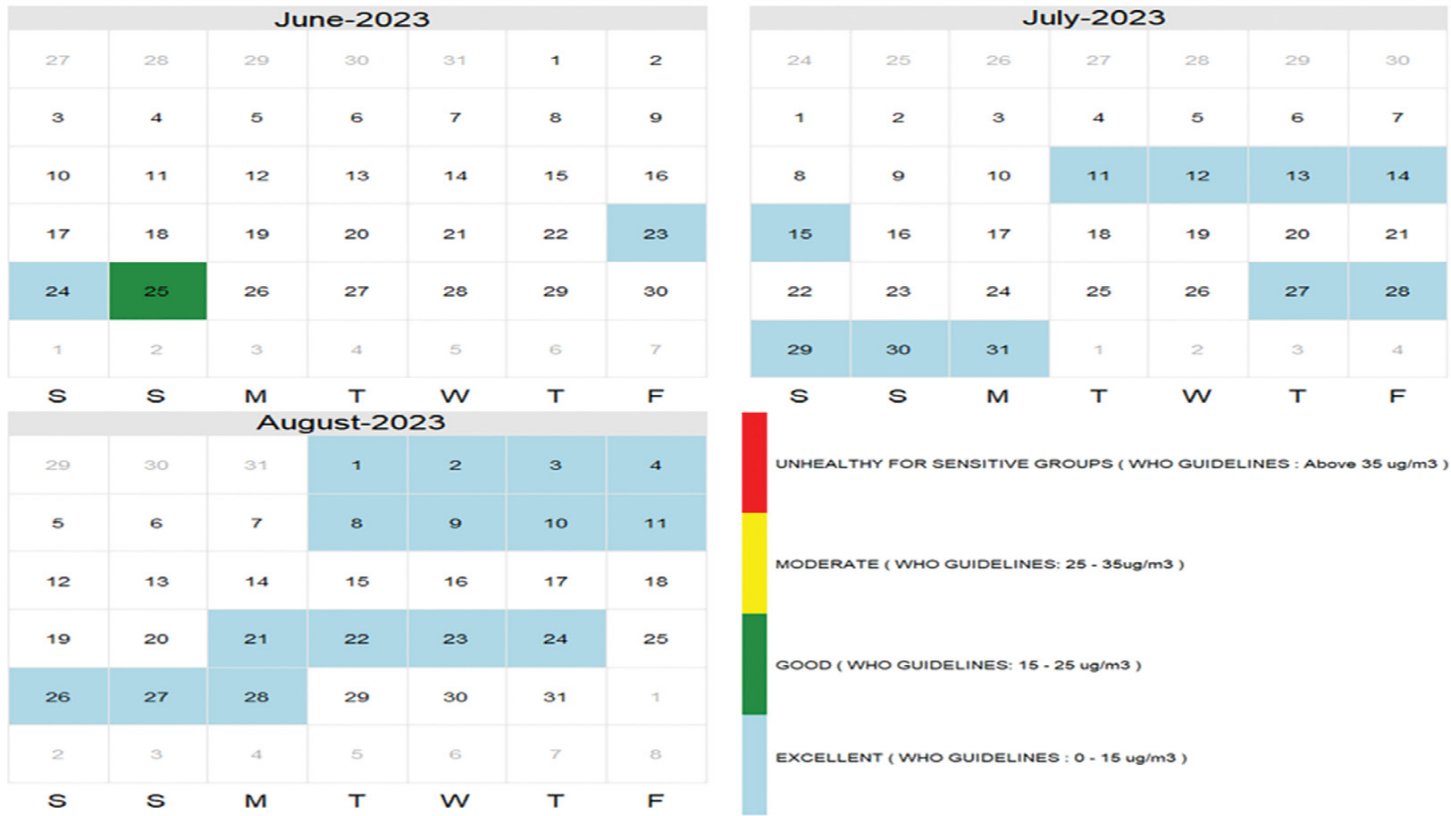
Calendar tracking three months’ activities.

*Household sensor data:* Data from the two households where sensors were successfully deployed illustrate the fragility of household-level monitoring systems. As shown in Figure 4, air quality levels fluctuated over the three-month period, with several “blue” days indicating acceptable air quality, while numerous gaps (white cells) reflect power outages during which sensors failed to transmit data.

**Figure 5:**
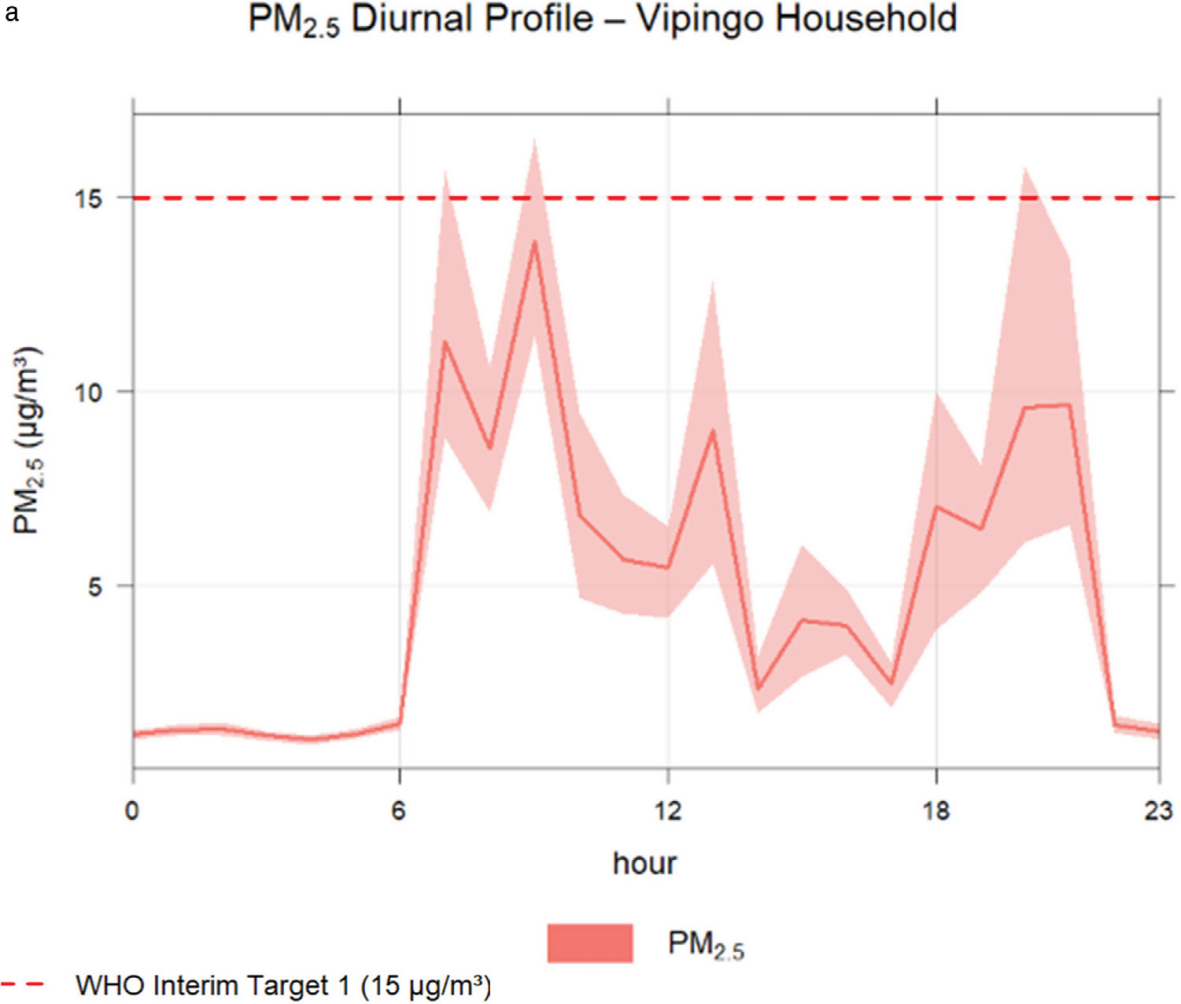

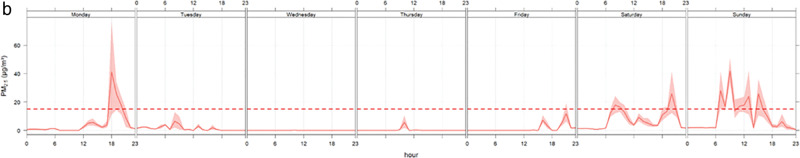
**(a)** One Monday hourly air quality period, and **(b)** presents weekly average scores over time.

In Figures 5a and b, we see one snapshot of Monday as an example of how much data varies over a 24-hour cycle and how weekly averages show the picture of air quality and spikes above the WHO PM_2.5_ permissible limit^4^ of 15 µg/m^3^.

*Air quality patterns:* Weekly averages indicate a deterioration in indoor air quality on Mondays, Saturdays, and Sundays, which may be attributable to increased household cooking activities when more family members are present. In contrast, midweek data (Wednesdays through Fridays) were incomplete due to frequent power outages that disrupted sensor functionality, as reflected by gaps in the dataset.

### Noise pollution

At Kangemi Health Center, the mean noise level was 52.2 dB, with a median of 53.5 dB. Recorded values ranged from a minimum of 28.3 dB to a maximum of 94.1 dB, highlighting considerable fluctuations. These levels far exceed the permissible limits established under Kenya’s Environmental Management and Coordination (Noise and Excessive Vibration Pollution) Regulations, which specify thresholds of 60 dB during daytime and 35 dB at night for health facilities. The findings underscore a persistently high and unregulated noise burden in a setting of heightened sensitivity.

### Environmental exposure survey

Survey results revealed 91.3% (*n* = 220) of respondents experienced extreme weather events. Lack of access to safe water was reported by 20.7% (*n* = 50), while 27.8% (*n* = 67) reported inadequate sanitation and hygiene facilities. Exposure to ambient outdoor air pollution was identified among 24.5% (*n* = 59), compared to 74.7% (*n* = 180) exposed to indoor air pollution. Noise-related exposure was reported by 17.4% (*n* = 42). Additionally, 93.8% (*n* = 226) of participants indicated they were affected by food insecurity, further compounding their vulnerability to environmental stressors.

### Mental health (MH) outcomes survey and exploratory inquiries

Eighty-three percent (*n* = 200) of participants were moderately to severely depressed as assessed on the Edinburgh Postnatal Depression Scale (EPDS) [32], 42.3% (*n* = 102) had moderate psychological distress as assessed on Kessler’s 10 scale [33], 84.2% (*n* = 203) had moderate perceived stress as assessed on Cohen’s perceived stress scale [40], 58.1% (*n* = 140) had mild anxiety as assessed on generalized anxiety disorder (GAD-7) [35]. Problematic family functioning accounted for 85.1% (*n* = 205) assessed via NIH PROMIS Measures. Elaborated mental health findings will be published in a separate manuscript.

### Journey mapping

The average blood pressure reading was 112.5/70.75, pulse was 91, heart rate was 84.8, and blood oxygen was 96%. Measurements were taken every five minutes along the way, and results were documented (see Table 2 and Supplementary eFigure 1). Descriptive statistics, mixed effect modeling, and time series analysis were used to analyze the data.

**Table 2:**
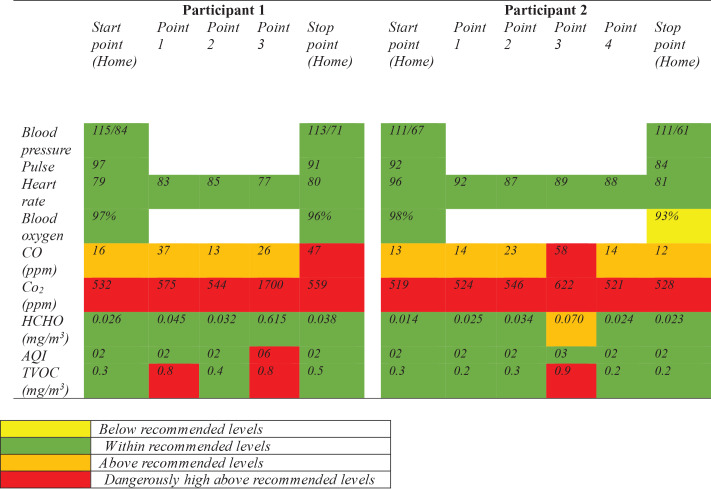
Journey mapping along routes perinatal adolescents take to school.

**Figure 6:**
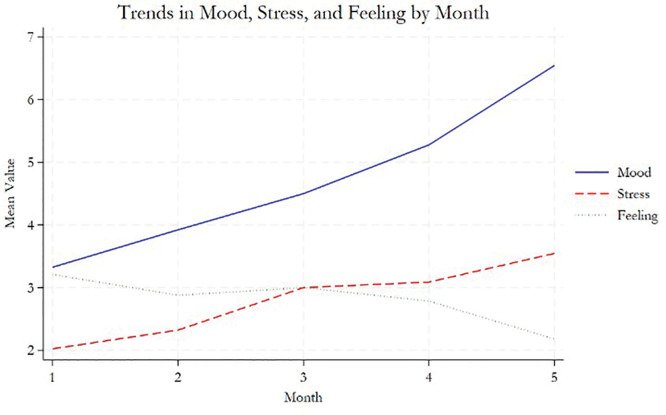
Trends in ecological momentary tracking of mood, stress, and feelings overtime.

In the household ecological momentary analysis, the mood, stress, and feelings ratings over five-month period are reported in Figure 6 and Supplementary eTable 1. We found that the living conditions of these participants were quite telling. The health outcomes of this group are impacted by socio-economic and structural conditions that determine where individuals are born, grow, live and age. Food insecurity is prevalent, leading to poor nutrition for adolescents and their children. Box 2 presents two case studies: one of participant 2 of journey mapping and one of an adolescent mother in Vipingo who participated in household sensor deployment, where we also carried out an ecological momentary exercise.

**Box 2. Case Studies from our Experience with Household Ecological Momentary Analysis and Journey Mapping**

**Case study 1: Sensor deployment in the household of an adolescent mother in vipingo, kilifi**

*Reflections on the process:* The adolescent mother of one child, who was 17 years old, was invited to participate, and she lived with her mother, who was single. The participant had dropped out of school in form two due to teenage pregnancy. The house was made of building blocks, the wall was not well-cemented, the roof was leaking, and the structure was incomplete as there was open space, making the sensor security an issue. The house was not well-lit, the door wasn’t strong, and the household was connected to the neighbor’s electricity, but the connection was poorly made with loose wires hanging within the walls, making it risky to operate. The house had only one socket that they used for charging their phones, and for the use of sensors, we had to add an extension cable. There was worry that the sensor’s continuous operation would be disrupted. The adolescent and her mother were taken through the sensor deployment, and they consented to participate. We provided them with data bundles and paid their electricity bills during this time. NWD, LK, and VN deployed the sensor after the initial difficulties.

*Reflections from CHWs:* The adolescent lived in a deprived environment, which compromised her privacy, since she had to share the little space and house with other family members. She was not entirely clear on the sensor’s purpose, despite confessing that she was oriented to its purpose at the beginning. However, she was grateful that when the sensor was in the household, the study paid for electricity tokens, which ensured constant connectivity when there was no power outage.

*Reflections from research assistant:* The structural conditions of the household were concerning, with deficiencies in general cleanliness, stability, and overall safety. The sensor installation faced additional risks as the dwelling was frequently accessed by extended family members and visitors, raising concerns about security and potential tampering. Frequent, unanticipated power outages further disrupted sensor operations, necessitating repeated field visits for inspection and troubleshooting.

Communication with the adolescent participant proved challenging. She did not own a personal phone and instead relied on her mother’s device, which was often unavailable during working hours. This constraint hindered timely follow-up and coordination of data collection activities. During the engagement, the adolescent’s mother reached out to members of the research team (LK and VN) to request support for her daughter’s return to school, underscoring the complex social and economic vulnerabilities intersecting with the technical challenges of household-level monitoring.

**Case study 2: Journey mapping with an adolescent mother in nairobi**

*Reflections on the process:* VN and EA led this inquiry in Kariobangi with an adolescent mother aged 18 years. The house was not well-ventilated or well-lit, and the electricity had to be used even during the day. During power outages, they used kerosene lamps or candles. The cooking was happening within the same room. From the outside of the house where the adolescent lived, there was running sewage, and the walkway was very narrow, muddy, and slippery. There was a busy tarmac road that led to the school where she studied, 100 meters away from her home. The road was quite busy, with pedestrians, motorbikes, and other vehicles with severe traffic emissions, and noise as well as general risk to safety as a pedestrian. The school was within a crowded residential space with lots of noise from the households and the main road. They came towards the health facility, walking through a busy road surrounding it, and the litter along the road was clearly visible.

*Reflections from CHWs*: The adolescent’s family faced socioeconomic hardships, poor living conditions, food insecurity, and poor sanitation, which affected her overall well-being. The house lacked proper ventilation and basic amenities and was crowded. As a young mother, these conditions augmented her challenges as she must care for her child in an unsafe environment to ensure healthy child development as well as her physical and mental health. Inadequate access to proper sanitation facilities poses a serious health risk, contributing to poor hygiene and a higher likelihood of disease transmission within the household, affecting the health and well-being of a young child. She faces financial instability, limiting her ability to meet basic needs for herself, her child, and her family as a whole since she lives with her mother and siblings. She struggles to afford essential items, such as food, hygiene products, and childcare necessities. This pressure is heightened by limited employment opportunities for her caregiver and support systems available to teenage mothers. The participant reported difficulty in consistently obtaining enough food for her family. Her mother’s limited financial capacity worsens the situation since she’s the sole breadwinner without a job or business. As a teenage mother facing a compounded set of challenges, in addition to the typical struggles of adolescence, she must navigate the responsibilities of motherhood within a difficult socioeconomic context. Her situation is further made complex by limited access to education and social support, which hinders her ability to improve her situation, putting her child’s future development at risk due to these hard conditions.

*Reflections from the research assistant:* Just the living conditions of the adolescent in a small and crowded house, occupied by her mother and other younger siblings portrayed a sense of struggle. Her mother, being a single parent and having to do casual jobs to earn a living and support the girl and her child as well, coupled with the burden of educating her daughter, looked overwhelmed with the responsibilities. Whenever the adolescent went to school, her mother had the added burden of taking the child to a daycare center so she could go to work; this meant a daily fee at the daycare center.

eTable 1 and Figure 4 present results of mixed-effect models for mood, stress, and feeling, and interaction effects over time. Supplementary File 1 provides more explanations of the analysis reported here. The positive coefficient for month (0.342) indicates mood increases by 0.3 units per month, on average. The mood of individuals tends to significantly decrease in the evening compared to the morning. The positive coefficient for month (0.288) indicates mood tends to increase by 0.3 units per month, on average. No significant change in stress levels is reported by time of day. On average, feeling tends to decrease over time (−0.148). This decline is consistent across all time categories but is modified by time of day. The feeling score is higher in the evening compared to the morning (0.549), suggesting that evenings are associated with better feelings irrespective of the time trend. In terms of interaction effects, the negative interaction term (−0.192) implies the rate of decline in feeling over time is greater in the evening compared to the morning. While the baseline feeling is higher in the evening, the negative effect of time on feeling is more pronounced in the evening.

## Discussion

The deployment of sensors and the associated sub-studies provided critical insights into the feasibility and challenges of integrating environmental monitoring into public mental health research. A key lesson was recognizing that environmental health issues must be studied across multiple scales—community, household, and health facility levels—since exposures and vulnerabilities vary substantially across these contexts.

These findings highlight the importance of embedding *implementation strategies*—defined as structured methods to enhance the adoption, execution, and sustainability of interventions—into climate and health initiatives. Effective mitigation and adaptation strategies require stakeholder engagement across the delivery system (e.g., health workers and community members), the support system (e.g., technical agencies and NGOs), and system leaders (e.g., policymakers and county authorities). Establishing interconnections among these actors is essential to enable routine monitoring, develop functional feedback mechanisms, and ensure that environmental and health data inform iterative quality improvement.

Crucially, integrating environmental monitoring with public health research has direct implications for *mental health outcomes*. Our findings suggest that exposure to poor air quality and unregulated noise is not only a physical health burden but also a psychosocial stressor that can exacerbate anxiety, mood disturbances, and reduced well-being. By capturing lived experiences through journey mapping and biomarker monitoring, the study demonstrated how environmental stressors intersect with socioeconomic vulnerabilities to shape mental health risks. Embedding these insights into urban planning, health policy, and climate adaptation strategies is therefore vital for building holistic resilience in vulnerable communities.

### Expanding understanding of adaptation and mitigation in the context of health systems strengthening

The study expands the conventional siloed understanding of adaptation and mitigation concepts used in environment and health programming. The adaptation framework demonstrates how health facilities and individuals respond to existing poor air quality and noise levels to reduce their impact on health, which will be the goal of future studies. As we emphasize strategies to cope with or manage the effects of air pollution and noise exposure, particularly on vulnerable populations, adaptation becomes a key priority. Our goal includes building resilience in health facilities to withstand the adverse effects of environmental hazards (e.g., through protective measures like air purification, soundproofing, or operational guidelines for healthcare delivery under poor air quality conditions). Future efforts extending lessons from this case study would be directed towards introducing these additional steps for building resilience at the system and system actors’ levels [41].

Mitigation here focuses on reducing sources or levels of air pollution and noise once these exposures are identified as hazardous. It is also relevant framing here as we emphasize the design and implementation of monitoring systems aimed at controlling or minimizing exposure to pollution and noise.

**Figure 7:**
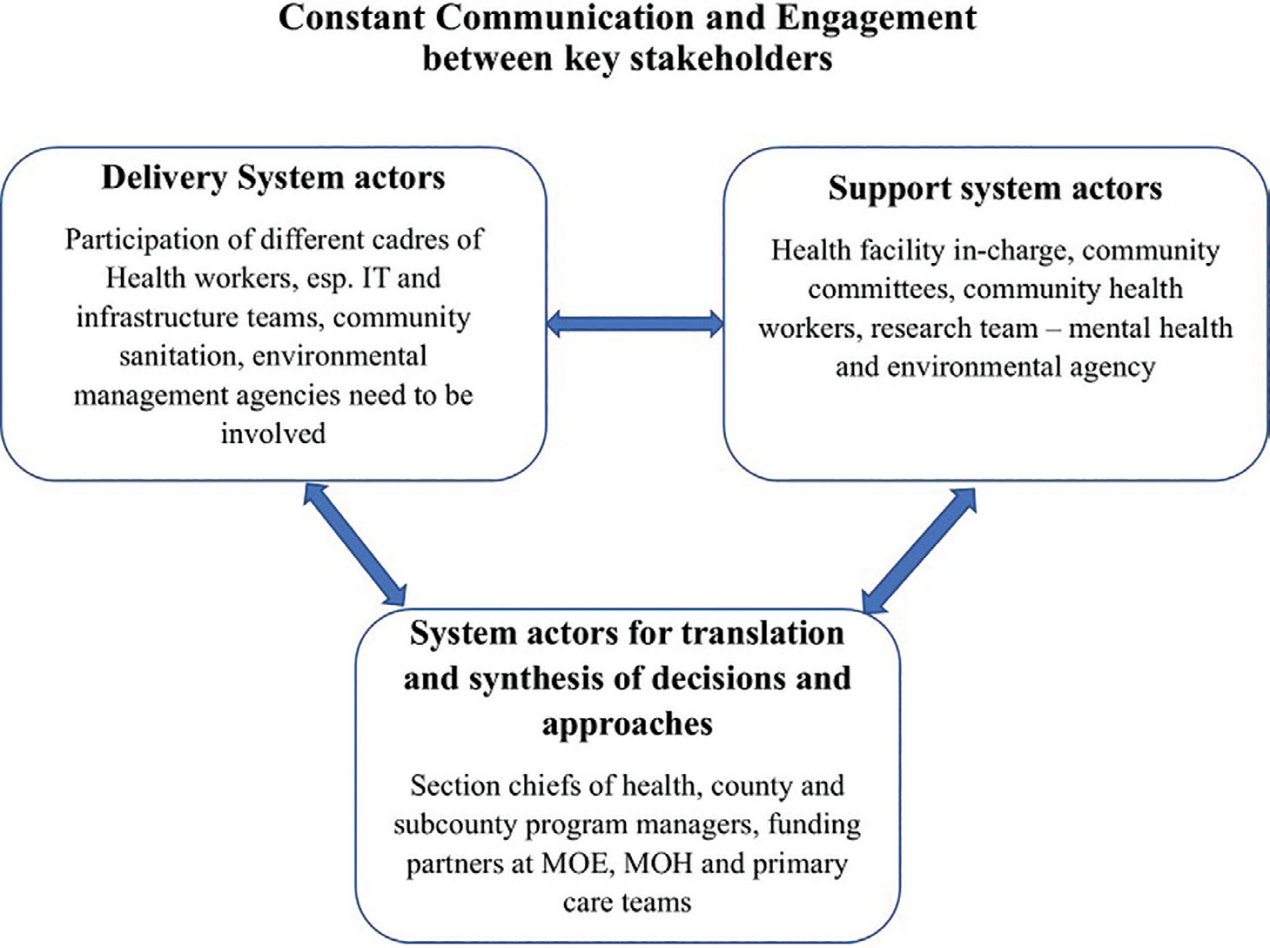
Adaptation and mitigation system actors.

A combined approach of adaptation and mitigation is relevant to our study, especially given that monitoring systems aim to reduce overall environmental pollution levels (e.g., through advocacy for cleaner practices or by generating data to inform policy interventions); mitigation is the better term. As our focus is also on how data can help design policies that tackle the root causes of pollution, mitigation continues as the focus particularly as monitoring systems are tools for reducing environmental risks and exposure. See Figure 7 which points to the synergies needed between Adaptation and Mitigation system actors. Communication across these two domains and stakeholders involved is critical.

### Climate adaptation and mitigation strategy

**Figure 8:**
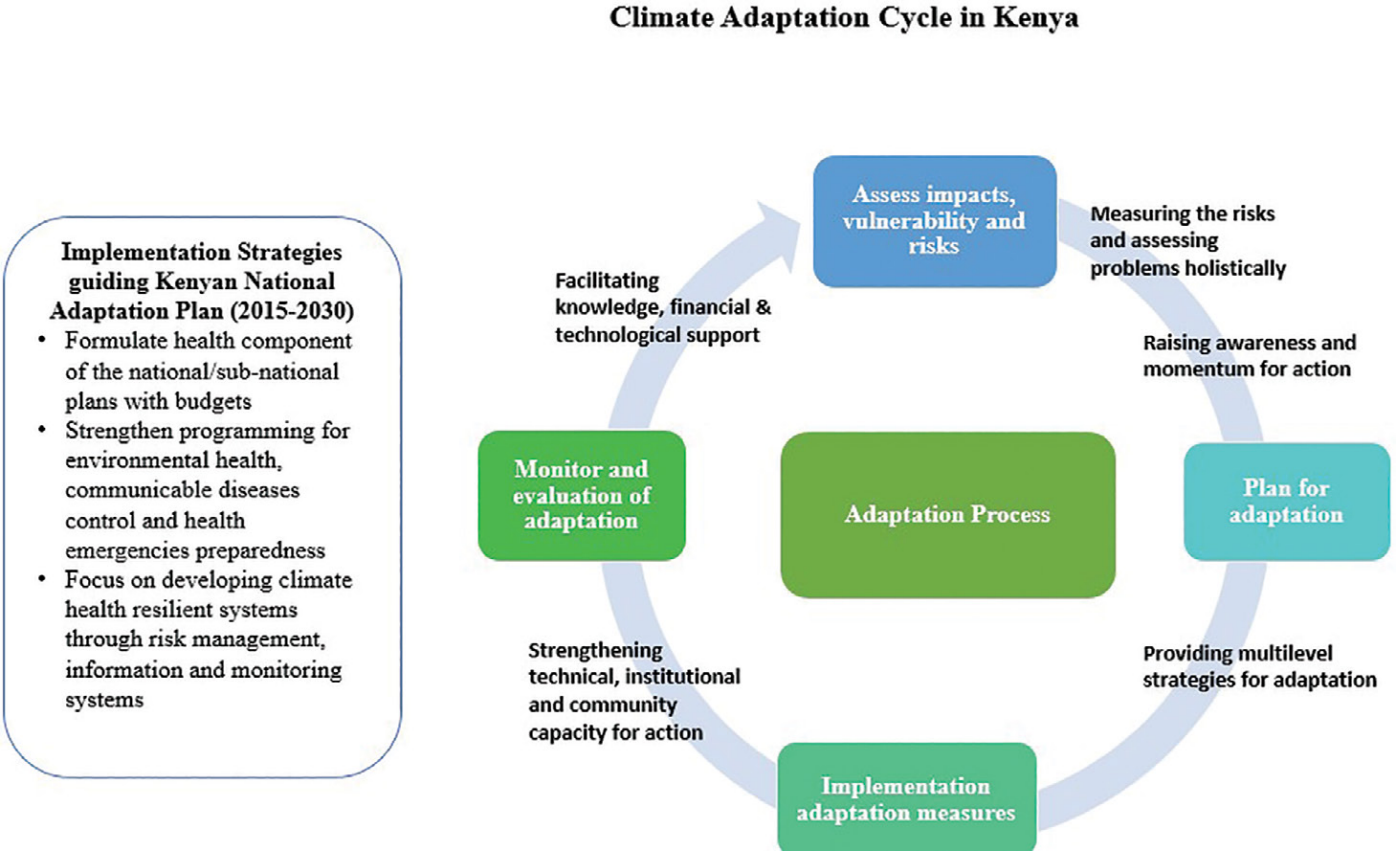
Climate adaptation cycle recommended by the Kenyan government.

Deploying air and noise sensors in index facilities and households with the help of a multidisciplinary team of environment, public, and mental health researchers backed by county health and Ministry of Environment officials was certainly the most important step of our strategy development. Information, knowledge, and awareness about sources, manifestations, and impacts of pollution and climate change-associated markers, like burning plastics, unclean energy, and fuel use, burning crops, excessive use of hazardous materials, and their leakage into water and soil, are poor within communities. These emerged as the exposures that the teams found the most challenging through conversations with different stakeholders. In their lives, non-climate impacts add greater stress and exacerbate vulnerabilities further. Adopting an adaptation and mitigation cyclic approach following UNFCCC would be a useful way of sequencing environmental and climatic health impacts (see Figure 8) [42]. In the context of this research, we learned that household and individual participant-level monitoring of exposures is challenging and needs a bigger capacity and investment. Leveraging CHWs, especially youth cadres within this workforce, can accelerate progress on monitoring and evaluation of these exposures and indicators. However, this group needs continuous training and support.

Our reflections on the overall process are presented in Table 3. We have divided these observations based on the different strategies we adopted. The strategies include capacity building, implementation process, integration, and dissemination.

**Table 3:** Areas of concern and efforts needed in the future for the refinement of strategies.

STRATEGY TYPE	SUB-COMPONENTS OF THE STRATEGY	STRATEGY ACTION	VALUE ADDED AND OUTCOMES	CHALLENGES ENCOUNTERED AND NEW ONES
**Capacity building** 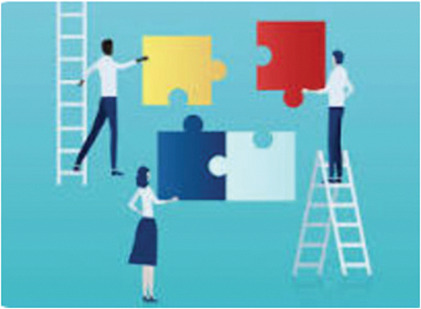	Building the team’s ability to understand measurement and monitoring of environmental exposures (train the trainer model) Identifying, training, and engaging health workers and lay CHWs who could monitor exposures	The research team needed a better grasp of environmental determinants that impact mental health/perinatal health outcomes. Knowledge of combined programming of mental health with environmental health areas for perinatal adolescents would advance holistic interventions	Learning Collaboration was developed that allowed a deeper sense of bidirectional learning between mental health and environmental teams. The importance of intervening at primary care and using the support of lay CHWs and facility staff for building in environmental health components for perinatal adolescents and other groups. Their own well-being in that environment became a question of concern apart from delivering community care	Teams had different priorities and expectations. Environmental teams wanted greater assistance with monitoring than anticipated. Having people attend to these issues within facilities at one time when sensors were installed was a challenge. Key people within facilities were busy/unavailable for continuous learning. Facility staff and lay CHWs did not have resources for community mobilization, and the study budget was limited
**Implementation process** 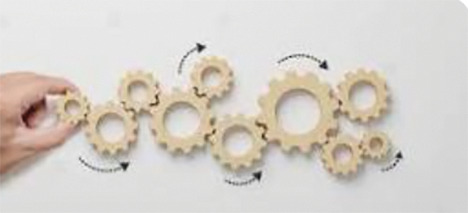	Deployment of sensors Education about their use and training for troubleshooting Engaging adolescents and the whole household/household head to install and maintain household-level sensors	Identification of appropriate sensors, technical choices for their selection, their right positioning, and additional tools/materials for their deployment took time and discussion. Research team members and health facility staff were identified for basic training on the sensors, additional gadgets (even those for protection), and communication around troubleshooting occurred throughout the period of sensor deployment. Basic environmental health education, engagement, and understanding the practical stressors of adolescents who volunteered their households for the deployment of sensors were actioned	The selection of the location of sensors helped in capturing the right exposure at the right clinics visited by perinatal populations There was a great deal of time spent training different members in understanding the deployment process, the need to monitor and ways to address problems and keep the sensors operational Identifying pregnant and parenting adolescents, and their caregivers to volunteer for the deployment of sensors was critical	There were times when power outages were experienced, and the health facility needed data bundles or internet services or when the sensors were switched off. Unavailability of reliable desktops at the health facilities to download data from noise sensors. The facilities in the Kenyan coastal county of Kilifi were far apart, and it took time to address issues. The staff did not engage too much once the gadget was deployed There were fights in the household over who “owned” the sensors; most households were poor and needed electricity and data support Households expected more financial support that could not be covered by the study. Someone had to be tasked to monitor the site adding to costs
**Integration** 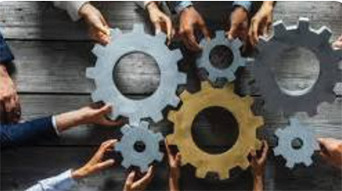	Addressing common factors Identifying environmental determinants of mental health	Combining the environmental and mental health survey for health facility and individual participants to map exposures and their impact on mental health. The study team had to identify areas such as sanitation, air quality, quality of life, food safety and quality, and home environment, which are linked to both environmental exposures and mental health	While combining the domains took time, it was beneficial to see how both areas could be brought together for exploration with perinatal adolescents. Distal and proximal risk factors identification using literature, in-country research & health/environmental policy-driven indicators was an exciting learning process	Polishing items for two distinct settings was not easy, Refining indicators for environmental domains specific to two landlocked and coastal ecosystems is a time-consuming process. Public health awareness of mental health is very low and still poor. Adding environment as a determinant is exciting but a challenging concept—the measurement of these exposures is still under-investigated, especially in LMICs
**Dissemination** 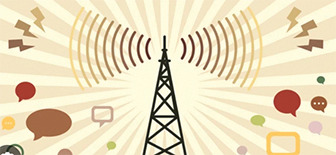	Consultative meetings on air and noise pollution quality Seeking facility and community feedback	Throughout the study and at the end, consultations were held on the health implications of air and noise pollution and the best practices for addressing these in primary care and community settings We sought feedback and participation from health facility actors to identify	The importance of shared learning and educating health service staff, community members, and the research community was felt in every engagement Community feedback provided new information on life conditions, problems with sanitation, water quality, and access to basic life amenities	Did not have adequate resources to provide in-depth training or education comprehensively, and to provide ready interventions. Resource and infrastructural issues are beyond the scope of any research, socio-structural problems like stigma and discrimination of adolescent mothers and those with mental illnesses need a wider national strategy and government investment
**Scale up** 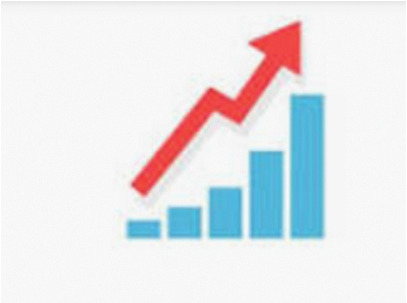	Engagement of stakeholders Willingness to share costs	Bringing together a well-represented collaborative of multidisciplinary researchers, clinicians, policymakers, and advocates from health and environmental backgrounds Agreement to share costs of sensors provided by UNEP—field deployment by the environmental agency and monitoring by the research team with partners and health facility ownership worked	Learning about the issues of environmental determinants of mental health was deep and informed by multiple perspectives Recognition of total investment needed for change and willingness to take some responsibility per entity, lots of cooperation	Sustained participation and attention towards integrated programming needs more conceptual harmonization and resources to strengthen the partnership. Resources may be needed and identified per entity to bring to the table for joint programming and intervention development, and their impact evaluation

The capacity building includes overall knowledge creation in linking themes of perinatal mental health to environmental exposure monitoring. The strategy included focusing on community health and primary care/facility-based health workers’ engagement and partnership on deployment of sensors, journey mapping, and carrying out quantitative and qualitative surveys. The implementation process acted as a learning strategy for all teams engaged in the work as we learned about the deployment of sensors—understanding different considerations for capturing indoor and outdoor air and noise pollution. A similar process was used to identify key environmental determinants of mental health. Integration involves connecting dots between two domains and talking to stakeholders about common risk factors. Dissemination is feeding back challenges, opportunities, lessons, and results of inquiries. Scaling up involves picking up monitoring strategies and broad lessons for further testing, such as maintaining and adding more facility-based sensors.

For routine monitoring of environmental exposures, it is necessary to have a basic healthcare and community health-based governance structure driven by evidence-based policies that prioritize integrated care. Strategic planning and collaboration with various stakeholders, especially with organizations affecting people’s health like water and sewage, nutrition, energy, and urban planning, are necessary to create a climate change-resilient health system [43].

### Forward-going monitoring and surveillance needed on the ground

Box 3 offers recommendations for improving monitoring. Surveillance of different exposures and population-level needs, especially of a vulnerable group like perinatal adolescents in their community settings, requires a multi-pronged approach covering coordination between the Departments of Health and Environment to provide electricity, water, and more resources to form a research laboratory within the primary care.

**Box 3. Recommendations for Improving Routine Monitoring of Environmental Health and Environmental Mental Health Outcomes**

**Table:** 

Coordinated environmental and health programming at county and sub-county levels
Deployment of air and noise pollution devices needs to also include other key exposures like soil, water, microplastics, etc., within the facility and key community spaces with a data dashboard for research, county/sub-county health decision-making, and key highlights shared with the facility
Identified mental health indicators, especially around perinatal adolescents, embedded within environmental health indicators
Trainer of trainers on a continuous basis for health facilities with lay health workers with strong advocacy to build a department for routine monitoring of exposures and outcomes at the sub-county level
Provision of electricity, internet, and backup support for data to review sensor information
Real-time monitoring feedback to feed into clinical, community, and local policy decision-making, including infrastructure costing and planning for the feedback process
Engaging communities to understand and address sources of pollution and poor air quality and developing guidelines for noise pollution regulation for health facilities

Real-time monitoring requires real-time feedback channels and mechanisms so that learnings and observations from the data can improve health decision-making and accelerate locally led adaptations around these adverse exposures.

## Conclusion

This study demonstrates that air and noise pollution exposures are not abstract environmental concerns but lived realities directly affecting community health, mental well-being, and health service delivery in Kenya. While journey mapping offered us an understanding of the “what” and “why” of these exposures and the experienced emotional and cognitive process including stress biomarker monitoring, the ecological momentary assessment explained how these psychological moments were tallying across time and within natural and built environmental contexts. Both inquiries were underpowered and meant to be exploratory in nature.

Our overall findings indicate that deploying sensors at household and health facility levels generated important experiential learning, both in terms of technical feasibility and social acceptability. Common challenges such as electricity outages, safety of devices, staff receptivity, and household dynamics, including domestic instability, complicated the implementation but also revealed the importance of tailoring monitoring strategies to communities’ socioeconomic and infrastructural realities. These insights suggest that adaptation and mitigation strategies must extend beyond purely technical solutions to include social and behavioral considerations.

Our results further highlight the interconnection between environmental exposures and mental health outcomes. Poor air quality, characterized by PM_2.5_ and PM_10_ exposure, has been linked globally to heightened risks of depression, anxiety, and cognitive decline, while noise pollution exacerbates stress, sleep disturbances, and psychosocial strain. In our context, household and facility-level monitoring revealed how cumulative exposures can act as silent contributors to mental health vulnerability, especially among young people and marginalized groups. This points to an urgent need for interventions that integrate environmental monitoring with psychosocial support and health service planning.

Looking ahead, several priorities emerge. First, robust time series analysis over longer periods is required to capture seasonal and spatial variations in exposure and to build predictive models that can inform health system preparedness. Second, scaling up deployment of low-cost sensors, coupled with investments in data infrastructure, will enable facility-level and community-level feedback loops that are essential for quality improvement. Third, integrating air and noise pollution into climate adaptation, urban planning, and public health strategies is necessary to address both immediate exposures and long-term resilience. Finally, collaboration across sectors—urban planning, transport, environment, energy, and health—will be crucial to mainstream equity in responses to environmental stressors.

In conclusion, addressing the dual burden of air and noise pollution on mental health requires a multi-systemic approach that bridges technical monitoring, community participation, and institutional capacity building across environment and health sectors. The way forward lies in embedding environmental exposures within public health priorities, strengthening data-driven planning, and empowering communities to lead adaptation and mitigation strategies. Doing so can create evidence-based pathways toward healthier, safer, and more resilient urban and peri-urban communities in Kenya and beyond.

## Notes

1 Process findings quantify a specific process or set of processes, flagging how well each performs.

2 Stress refers to a mental or emotional strain due to some environmental or interpersonal adversity or disturbance.

3 A particular feeling or state of mind at a particular point in time.

4 According to the World Health Organization (WHO) Air Quality Guidelines, the AQG level provides an evidence-informed derivation associated with the exposure to pollutants. In our case, PM, particulate matter (PM_2.5_) is regarded as a pollutant with a direct impact on health. The exceedance of PM_2.5_ over 24 hours should not be above 15 µg/m^3^, which is evaluated based on the 99th percentile (3–4 exceedance days per year), and the annual exceedance should not be above 5 µg/m^3^.

## Additional Files

The additional files for this article can be found as follows:

Supplementary File 1. Figure S1 and Table S1. Results for ecological momentary analysis.

Supplementary File 2. Air pollution sensors.
