# Supplementary material for: Air–Noise Pollution Linkages: Testing Innovative Community-Based Adaptation and Mitigation Strategies in Kenya
Source: Ann Glob Health. 2025 Oct 28;91(1):74. doi: 10.5334/aogh.4750 (PMC12577548; doi:10.5334/aogh.4750)
Supplement: Supplementary File 1. — Figure S1 and Table S1. Results for ecological momentary analysis. [file agh-91-1-4750-s1.pdf]

Supplementary Tables and Figures

Results for ecological momentary analysis

eTable 1 : Mixed effect models for mood, stress and feeling

**Variable**	**Category**	**Mood**	**Stress**	**Feeling**
		**Model 1 Coef. (SE) [95% CI], p**	**Model 2 Coef. (SE) [95% CI], p**	**Model 3 Coef. (SE) [95% CI], p**
**Time of the day**	Morning	Ref.	Ref.	Ref.
	Afternoon	-0.882 (0.596) [-2.050, 0.285], p=0.139	-0.257 (0.217) [-0.681, 0.168], p=0.236	0.389 (0.266) [-0.132, 0.909], p=0.143
	Evening	-1.243 (0.617) [-2.452, -0.034], p=0.044	-0.113 (0.224) [-0.552, 0.327], p=0.615	0.549 (0.275) [0.010, 1.088], p=0.046
**Time in Months**	Time	0.342 (0.141) [0.065, 0.619], p=0.015	0.288 (0.051) [0.188, 0.388], p<0.001	-0.148 (0.063) [-0.272, -0.024], p=0.019
**Time of the day × Month**	Morning	Ref.	Ref.	Ref.
	Afternoon	0.341 (0.203) [-0.058, 0.739], p=0.094	0.078 (0.074) [-0.067, 0.223], p=0.292	-0.130 (0.091) [-0.307, 0.048], p=0.151
	Evening	0.387 (0.206) [-0.018, 0.791], p=0.061	0.046 (0.075) [-0.101, 0.193], p=0.543	-0.192 (0.092) [-0.372, -0.012], p=0.037
**Constant**		3.797 (0.579) [2.662, 4.932], p<0.001	1.957 (0.185) [1.594, 2.320], p<0.001	3.107 (0.282) [2.555, 3.659], p<0.001
**Random-effects parameters**	var(_cons)	0.484 (0.445) [0.080, 2.936]	0.035 (0.036) [0.005, 0.268]	0.134 (0.114) [0.025, 0.709]
	var(Residual)	2.605 (0.214) [2.217, 3.060]	0.345 (0.028) [0.293, 0.405]	0.517 (0.043) [0.440, 0.608]

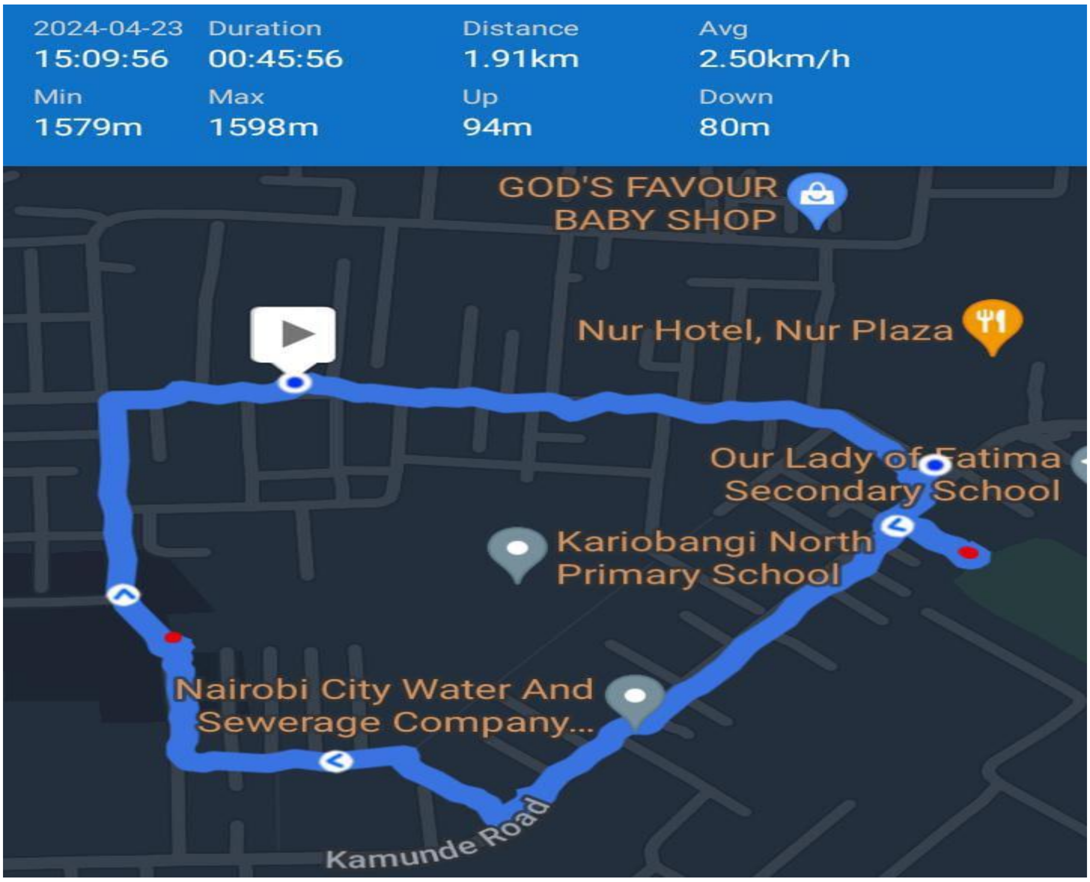

eFigure 1: Track followed by participant 2 in journey mapping
