# Supplementary material for: Air–Noise Pollution Linkages: Testing Innovative Community-Based Adaptation and Mitigation Strategies in Kenya
Source: Ann Glob Health. 2025 Oct 28;91(1):74. doi: 10.5334/aogh.4750 (PMC12577548; doi:10.5334/aogh.4750)

Supplementary data analysis information

Data Processing and Cleaning

Loading and Inspecting the Data

The first step in the analysis involves loading the data and inspecting its structure using R. We used the read.csv() function to import the dataset, and functions like str() and summary() were employed to check the overall structure and summary statistics of the data.

Handling Missing Data

Due to frequent power outages in the household where the sensor was placed, significant periods of missing data were encountered. Missing data was identified using the is.na() function in R, and these gaps were removed for a cleaner analysis. However, removing the data entirely could reduce the completeness of our analysis, so alternative approaches such as imputation methods could be explored in future work.

Statistical Methods Used in the Analysis

Descriptive Statistics

We computed basic descriptive statistics to understand the distribution of PM2.5 concentrations. This included daily averages, minimums, and maximums of PM2.5 levels, calculated using the dplyr package.

Exceedance Analysis

We also analyzed the number of days where the PM2.5 concentration exceeded the World Health Organization (WHO) guideline of 15 µg/m³ for the 24-hour average. This helped us evaluate the extent of exposure to unhealthy levels of indoor air pollution.

Challenges in the Analysis: Missing Data

One of the main challenges in this analysis was the presence of missing data due to frequent power outages at the household where the air quality sensor was installed. Missing data introduces biases and limits the ability to conduct a full analysis of the pollution levels.

Methods for Addressing Missing Data

In this analysis, missing data was simply removed from the dataset. However, for future work, several strategies can be explored to deal with missing data:

- **Interpolation**: Use linear interpolation to estimate missing PM2.5 values based on nearby data points.
- **Imputation**: Implement statistical imputation techniques like regression imputation or k-nearest neighbors (KNN) to fill gaps in the data.
- **Using External Data**: Correlate the data with external air quality datasets (e.g., from nearby weather stations or other sensors) to approximate missing values.

Despite these challenges, the overall trends in the data were still apparent, and key insights regarding PM2.5 concentration were obtained.

Conclusion

The analysis of indoor PM2.5 levels using R revealed important findings about the household air quality. While power outages led to gaps in the data, future efforts could explore ways to mitigate missing data through interpolation or external data integration. The visualizations and statistical analyses provide valuable insights into the time periods of highest pollution, which can inform mitigation strategies to protect the health of household occupants, particularly children and individuals with respiratory conditions.

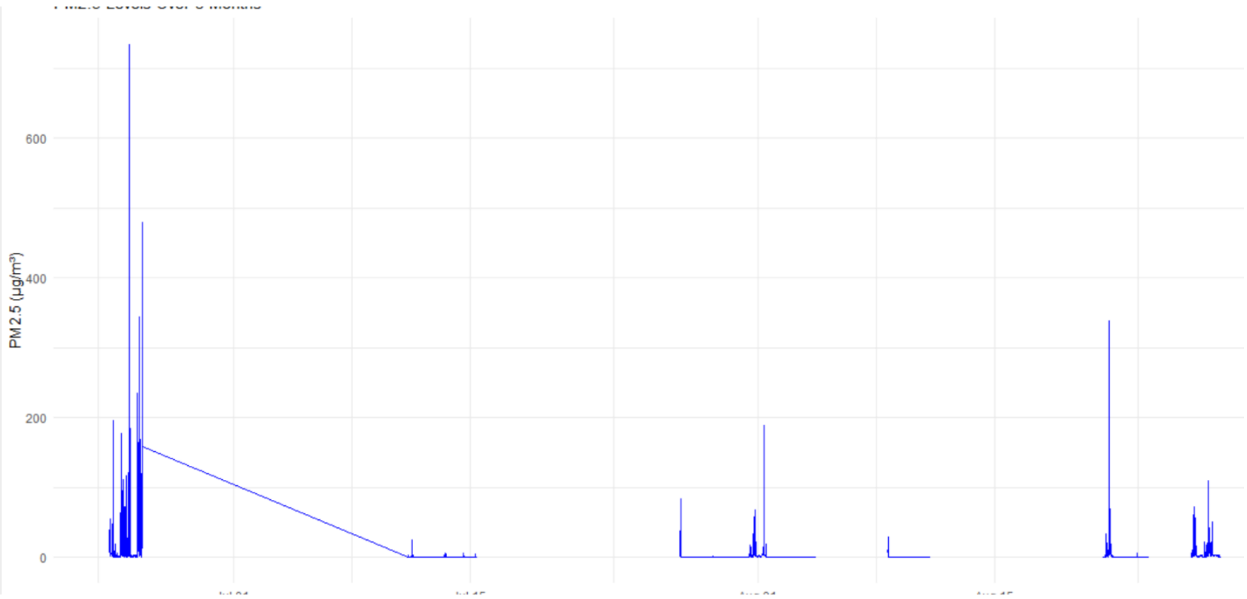

Supplement: Supplementary File 2. — Air pollution sensors. [file agh-91-1-4750-s2.pdf]
